# Very Rare Presentation of Hernia of Umbilical Cord

**DOI:** 10.21699/jns.v6i2.490

**Published:** 2017-04-15

**Authors:** Aditya Pratap Singh, Ramesh Tanger, Vinay Mathur, Arun Kumar Gupta

**Affiliations:** Department of Pediatric Surgery, SMS Medical College Jaipur, Rajsthan, India

**Dear Sir**

We read with interest review article entitled “Distinct Presentations of Hernia of Umbilical Cord” published in volume 5 of Journal of neonatal Surgery. [1] We also managed a case of hernia of umbilical cord (HUC) presented with bowel evisceration and mimicking gastroschisis.

A baby girl, weighing 2.5 kg, presented on day 1 of life with a bowel lying outside the abdominal cavity. She born at 38 weeks gestation by spontaneous vaginal delivery. Antenatal scans at 13 and 17 weeks detected gastroschisis. Her APGAR score was satisfactory. On examination, the bowel was eviscerated outside the abdominal cavity through the small defect at the lateral side of the base of umbilicus with hernia of umbilical cord (Fig.1). Exploratory laparotomy showed part of ascending colon with the caecum and appendix as a content of the HUC. The eviscerated bowel was atretic ileum with thickened peel, matted, shortened, oedematous and swollen bowel loops. There were entry and exist level atresias present at the level of ascending colon and jejunum. There was non-rotation of the midgut. The eviscerated bowel with atretic segment was resected and jejuno-colic anastomosis formed. On postoperative day 6, she was on full feeds. Baby was discharged on the 8th postoperative day.

Hernia of umbilical cord (HUC) with evisceration of the bowel is extremely rare. [2] During physiological herniation of bowel, the umbilical coelom may get ruptured leading to evisceration of herniated bowel. [3] After evisceration, the bowel loops float freely in the amniotic cavity as happens in gastroschisis. If no further devastating events take place, these patients are born with eviscerated bowel from one side of umbilical cord. The bowel and mesentery look edematous, swollen and is covered by a thick fibrinous peel; its mimics as gastroschisis [2] on initial inspection. It is usually associated with entry/exit level atresias [2]. All these findings were present in the index case.

**Figure:**
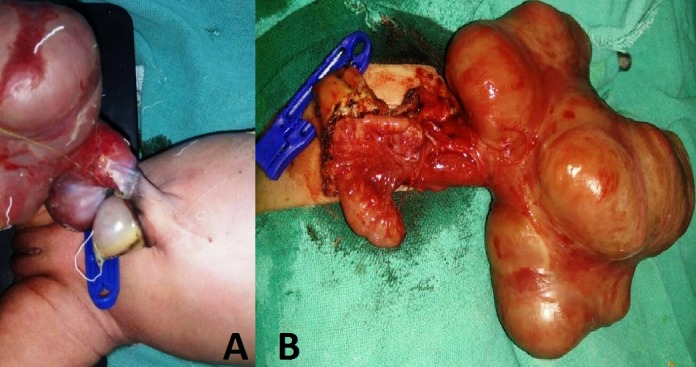
Figure 1: A) Showed HUC with eviscerated bowel. B) Showed atretic colon with caecum and appendix.

## Footnotes

**Source of Support:** Nil

**Conflict of Interest:** None
